# Case Report: Leaflet Thrombosis After Transcatheter Aortic Valve Replacement With Worsening Heart Failure—A Successful Resolution Using Non-vitamin K Antagonist Oral Anti-coagulant

**DOI:** 10.3389/fcvm.2021.731427

**Published:** 2021-12-17

**Authors:** Kae-Woei Liang, Chu-Leng Yu, Wei-Wen Lin, Wen-Lieng Lee

**Affiliations:** ^1^Cardiovascular Center, Taichung Veterans General Hospital, Taichung, Taiwan; ^2^Department of Medicine, School of Medicine, National Yang Ming Chiao Tung University, Taipei, Taiwan; ^3^Department of Surgery, School of Medicine, National Yang Ming Chiao Tung University, Taipei, Taiwan; ^4^Department of Life Science, Tunghai University, Taichung, Taiwan; ^5^Department of Medicine, China Medical University, Taichung, Taiwan

**Keywords:** aortic valve stenosis (AS), case report, leaflet thrombosis, transcatheter aortic valve replacement (TAVR), transcatheter heart valve, non-vitamin K antagonist oral anti-coagulant

## Abstract

**Background:** Transcatheter aortic valve replacement (TAVR) is indicated for treating symptomatic severe aortic valve stenosis (AS) with intermediate-to-high surgical risks. Few reports are available on managing leaflet thrombosis after TAVR with worsening heart failure.

**Case Summary:** A 77-year-old man with severe AS and coronary artery disease (CAD) received a successful TAVR with Edwards Sapien 3 valve. A year later, the patient developed a worsening heart failure with pulmonary edema, new-onset atrial fibrillation (Af), an increase in mean trans-aortic valve pressure gradient to 48 mmHg, worsening mitral regurgitation (MR), and pulmonary hypertension (PH). The response of the patient to intravenous diuretics and inotropic treatments was poor. Multi-slice CT (MDCT) revealed hypo-attenuated thrombus and thickened transcatheter heart valve leaflets. A non-vitamin K antagonist oral anti-coagulant (NOAC) was added to treat the new-onset Af and leaflet thrombosis on top of the con-current single antiplatelet for CAD. A series of follow-up echocardiograms showed a progressive decrease in trans-aortic valve pressure gradient to 17 mmHg and reductions in MR and PH. Three months after the NOAC treatment, MDCT revealed the resolution of hypo-attenuated thrombus and thickened leaflets. Symptoms of heart failure were also improved gradually.

**Discussion:** Worsening heart failure or an increase in trans-aortic valve pressure gradient after TAVR warranted further MDCT studies. Leaflet thrombosis can be resolved after using NOAC as in our present case.

## Introduction

Transcatheter aortic valve replacement (TAVR) is indicated for treating symptomatic severe aortic valve stenosis (AS) with intermediate-to-high surgical risks (1). The latest guideline supports the use of TAVR for symptomatic severe AS in patients ≥65 years of age (2). Current guidelines also recommend a 3–6 months use of dual antiplatelet agents and life-long aspirin treatment following TAVR (2).

Structural valve deterioration (SVT) is defined as an increase in mean gradient ≥10 mmHg or a new mean gradient ≥20 mmHg (1, 3). Leaflet thrombosis is one of the etiologies of SVT after TAVR and could be observed and detected by hypo-attenuated leaflet thickening (HALT) and reduced leaflet motion (RLM) using multi-slice CT (MDCT) (3, 4). No consistent outcome of subclinical leaflet thrombosis is known. Most reports did not correlate subclinical leaflet thrombosis on MDCT with a worse outcome of SVT (5). However, some patients might suffer from clinical leaflet thrombosis with evidence of increased pressure gradient of the transcatheter heart valve (THV), valve stenosis, worsening heart failure that warrant an anti-coagulant therapy (6, 7).

Herein, we reported a 77-year-old man with severe AS and coronary artery disease (CAD). The patient had previously received TAVR with Sapien 3 valve (Edwards Lifesciences, Irvine, CA, USA). A year later, the patient developed leaflet thrombosis of THV, a new onset of atrial fibrillation (Af), and worsening heart failure. The patient was successfully treated with a non-vitamin K antagonist oral anti-coagulant (NOAC) on top of the concurrent single antiplatelet medication.

## Case Description

**Figure 1:**
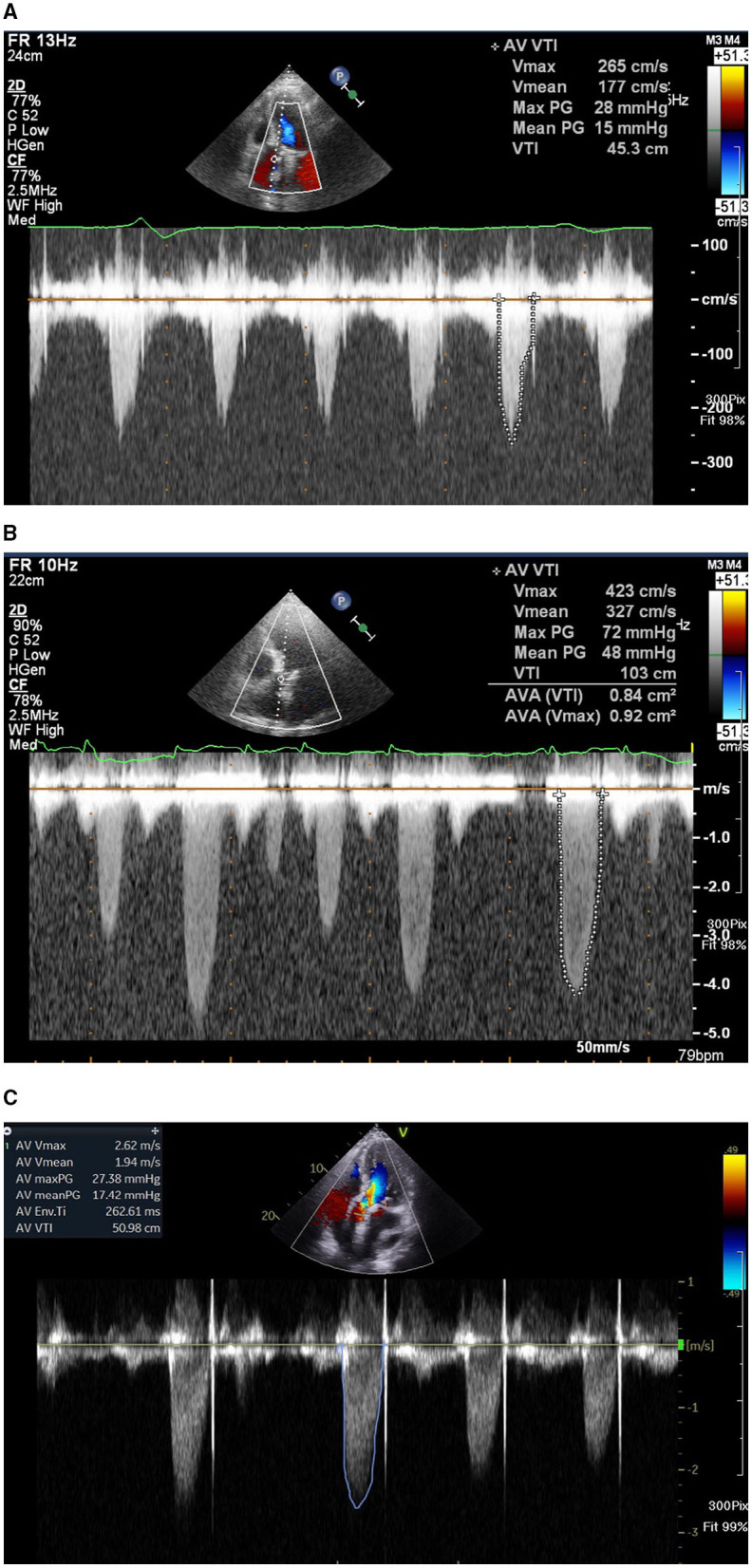
**(A)** Echocardiogram showing a baseline trans-aortic valve mean pressure gradient of 15 mmHg 1 day after trans-aortic valve replacement (TAVR). **(B)** One year later, the patient developed a worsening heart failure, new onset of atrial fibrillation, and an increase of echocardiography-derived mean trans-aortic valve pressure gradient of 48 mmHg. **(C)** After 3-month treatment with non-vitamin K antagonist oral anti-coagulant, echocardiogram showing a mean trans-aortic valve pressure gradient of 17 mmHg.

**Figure 2:**
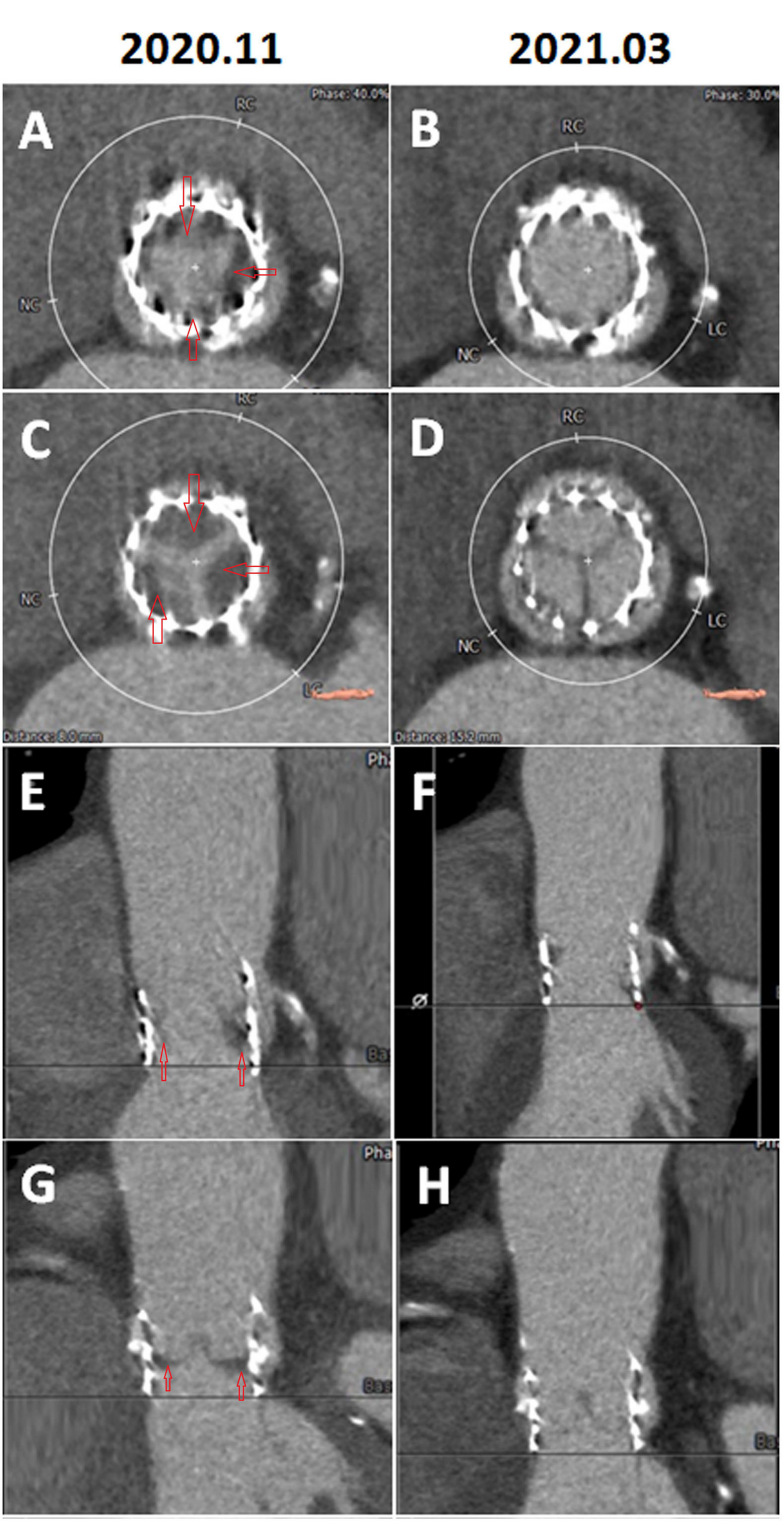
**(A)** Systolic phase, two-dimensional (2D), short-axis view showing a hypo-attenuated leaflet thickening (HALT) suggestive of thrombus involving the base of leaflets (red arrows). **(B)** Systolic phase, 2D, short-axis view showing the resolution of HALT after 3-month of anti-coagulant therapy. **(C)** Diastolic, 2-D, short-axis view showing HALT suggestive of thrombus involving the base of leaflets (red arrows). **(D)** Diastolic phase, 2D, short-axis view showing resolution of HALT after 3-month of anti-coagulant therapy. **(E)** Systolic phase, 2D, long-axis view showing HALT suggestive of thrombus involving the base of leaflets (red arrows) and reduced systolic leaflet motion. **(F)** Systolic phase, 2D, long-axis view showing resolution of HALT and resumed normal systolic leaflet motion after 3-month of anti-coagulant therapy. **(G)** Diastolic phase, 2D, long-axis view showing HALT suggestive of thrombus involving the base of leaflets (red arrows). **(H)** Diastolic phase, 2D, long-axis view showing the resolution of HALT after 3-month of anti-coagulant therapy. RC, right coronary cuspid; LC, left coronary cuspid; NC, non-coronary cuspid.

A 77-year-old man had CAD and underwent percutaneous coronary intervention (PCI) in November 2017 and October 2019. The patient had severe AS with trans-aortic valve mean pressure gradient of 47 mmHg and received TAVR with a 29 mm Edwards Sapien 3 valve uneventfully in November 2019. A day after TAVR, echocardiographic-derived mean trans-aortic valve pressure gradient of the patient was 15 mmHg (Figure 1A). Dyspnea was improved, and the patient had good exercise tolerance thereafter. One year later (October 2020), the patient suffered from worsening heart failure with pulmonary edema. ECG of the patient revealed a new-onset Af and the echocardiogram disclosed an increased mean THV pressure gradient to 48 mmHg (Figure 1B), worsening mitral regurgitation (MR), and pulmonary hypertension (PH). Response of the patient to standard heart failure treatment, such as intravenous inotropics and diuretics, was poor. MDCT revealed HALT and RLM (Figures 2A,C,E,G). On the top of the concurrent single antiplatelet medication for CAD of the patient, a NOAC (rivaroxaban) was added for the new-onset Af and leaflet thrombosis. A series of follow-up echocardiograms within 3 months showed a progressive drop in trans-aortic valve pressure gradient to 17 mmHg (Figure 1C), together with reduced MR and PH. MDCT showed resolution of HALT and RLM (Figures 2B,D,F,H) 4 months after NOAC treatment. Heart failure symptoms improved gradually but Af persisted. Unfortunately, the patient had a passage of tarry stool and drop of hemoglobin to 7.2 mg/dl in the fifth month after concomitant use of clopidogrel and rivaroxaban. The patient received a therapeutic endoscope, proton pump inhibitors, and blood transfusion for stopping the upper gastrointestinal bleeding. The patient discontinued clopidogrel but kept on using rivaroxaban and was free from heart failure symptoms and bleeding events thereafter.

## Discussions

Multi-slice CT has been used to detect subclinical leaflet thrombosis of THV since 2015 (3, 4). The PORTICO IDE trial reported that 1 month after TAVR, RLM and HALT of THV were present in 22 of 55 patients (40%) (4). However, the finding was deemed asymptomatic and subclinical as no difference was found in mean THV pressure gradients between those with or those without RLM (5). MDCT findings of PARTNER 3 CT sub-study revealed a similar high incidence of HALT in TAVR and surgical aortic valve replacement (SAVR) after a period of 1 year (27.5 vs. 20.2%, *p* = 0.19) (5).

Echocardiographic evaluation of HALT or RLM is frequently more challenging due to acoustic shadowing and ring-down artifacts arising from the valve struts. Previous publications on the incidence of THV thrombosis were detected by echocardiography after TAVR ranged from 0.6 to 7.6% (3, 8). Thus, the role of echocardiography is aimed at detecting an abnormal increase of THV mean pressure gradient and MDCT should be the first option for evaluating HALT and RLM. Our case had a series of transthoracic echocardiography showing a normal trans-THV pressure gradient immediately post-TAVR, followed by an abnormal increase of pressure gradient after leaflet thrombosis and returning normal gradient after NOAC use (Figure 1). The patient also had a transesophageal echocardiography (TEE) during the clinical suspicion of leaflet thrombosis. However, due to morbid obesity and poor acoustic window, it is difficult to evaluate HALT or RLM by TEE.

However, a minority of patients might suffer from clinical leaflet thrombosis with symptoms of heart failure and an increased THV pressure gradient. One TAVR MDCT study revealed THV thrombosis in 28 of 405 (7%) patients (9). Among them, 23 patients had subclinical THV thrombosis, whereas 5 patients experienced clinically overt obstructive THV thrombosis [i.e., 18% of leaflet thrombosis cases (5/28) or 1.2% of total cases (5/405)] (9). Based on multivariable analysis, a THV of 29-mm (relative risk 2.89) and no post-TAVR warfarin treatment (relative risk 5.46) independently predicted the THV thrombosis (9). Treatment with warfarin effectively reverted THV thrombosis and normalized THV functioning in 85% of patients, as documented by follow-up TEE and MDCT (9). Another meta-analysis reported a 1.2% prevalence of clinical THV thrombosis after TAVR, which was associated with the increased incidence of stroke but not with mortality (7). Our patient had a 29 mm Sapien 3 valve and developed clinical leaflet thrombosis 1 year after TAVR with elevated THV pressure gradient and evidence of HALT and RLM in MDCT and with worsening heart failure with pulmonary edema. The patient had a satisfactory recovery from clinical leaflet thrombosis after NOAC treatment as evidenced by the resolution of HALT and RLM in MDCT (Figure 2) and normalization of THV pressure gradient based on echocardiogram (Figure 1).

Current guidelines for patients with low risk of bleeding and without additional risk factors for thromboembolism recommend dual antiplatelet use for 3–6 months after TAVR (2). In patients without established indications for oral anticoagulation, a treatment strategy after TAVR, such as rivaroxaban, at a dose of 10 mg daily was associated with a higher risk of death or thromboembolic complications and a higher risk of bleeding when compared with an antiplatelet-based strategy (10). In contrast, for subjects with concomitant indications for using oral anticoagulants (mostly due to Af) after TAVR, NOAC was equal to warfarin in safety and survival outcome in registry data and case series (11, 12). Our case followed these recommendations using dual antiplatelet right after TAVR for 6 months, followed by single antiplatelet use. NOAC was used after detecting the onset of Af and THV leaflet thrombosis.

Regarding the choice between NOAC and warfarin for treating THV clinical leaflet thrombosis after TAVR, most reports used either warfarin or NOAC, both of which resolved HALT and showed clinical improvements (4, 5, 13). In our case, the patient had a concomitant new-onset Af and clinical THV leaflet thrombosis. Current guidelines for patients with a bioprosthetic valve and new-onset Af recommend using warfarin during the initial 3 months (2). Despite the recommendation, we had considered convenience and ease of administration and chose NOAC instead. It was able to resolve HALT and RLM with an improved THV pressure gradient.

In conclusion, we proposed that patients with worsening heart failure after TAVR should be evaluated with the echocardiogram for THV pressure gradient and MDCT for HALT and RLM. Findings would allow differential diagnosis of clinical leaflet thrombosis. Successful resolution can be achieved with NOAC use as demonstrated in our case.

## Data Availability Statement

The original contributions presented in the study are included in the article/Supplementary Material, further inquiries can be directed to the corresponding authors.

## Ethics Statement

Written informed consent was obtained from the individual(s) for the publication of any potentially identifiable images or data included in this article.

## Author Contributions

K-WL, C-LY, W-WL, and W-LL: case treatment, manuscript preparation, diagnosis, and literature review. All authors contributed to the article and approved the submitted version.

## Conflict of Interest

The authors declare that the research was conducted in the absence of any commercial or financial relationships that could be construed as a potential conflict of interest.

## Publisher's Note

All claims expressed in this article are solely those of the authors and do not necessarily represent those of their affiliated organizations, or those of the publisher, the editors and the reviewers. Any product that may be evaluated in this article, or claim that may be made by its manufacturer, is not guaranteed or endorsed by the publisher.
